# Perceived Stress among Healthcare Students and Its Association with Anxiety and Depression: A Cross-Sectional Study in Saudi Arabia

**DOI:** 10.3390/healthcare11111625

**Published:** 2023-06-01

**Authors:** Monira Alwhaibi, Ameerah Alotaibi, Budoor Alsaadi

**Affiliations:** 1 Department of Clinical Pharmacy, College of Pharmacy, King Saud University, Riyadh 11149, Saudi Arabia; 2 Medication Safety Research Chair, College of Pharmacy, King Saud University, Riyadh 11149, Saudi Arabia

**Keywords:** perceived stress, healthcare students, anxiety, depression

## Abstract

**Introduction**. Healthcare students are subjected to high-stress levels due to different academic, social, and financial stressors. Persistent and severe stress might predispose students to depression and anxiety. Therefore, this research aims to investigate the level of perceived stress among healthcare students and its relation to anxiety and depression. **Methods**. A prospective cross-sectional study using a validated questionnaire was conducted among healthcare students in Saudi Arabia. The 14-item Perceived Stress Scale (PSS) was used to evaluate perceived stress, and depression and anxiety were measured using the Hospital Anxiety and Depression Scale (HADS). All statistical analyses were carried out using PSPP Statistical Analysis Software version 1.2.0. **Results**. A total of 701 respondents participated in this study. The average age of the students was 20.9 years old, and 59.3% were female. Almost three-quarters of students perceive themselves as stressed. Around two-thirds were categorized as having borderline/cases of depression or anxiety. Perceived stress was four times more likely among students with cases of anxiety [Adjusted Odds Ratio (AOR) = 4.83; 95% CI 2.89, 8.06], depression [AOR = 4.79; 95% CI 2.68, 8.53] as compared with those without these conditions. **Conclusions**. Stress is highly prevalent among healthcare students, and it is strongly associated with female gender and students’ anxiety and depression. Therefore, the mental health of healthcare students is an essential factor affecting perceived stress and at-risk individuals. Therefore, preventative mental health interventions targeting healthcare students are necessary to help improve mental health and cope with stressors in academic education.

## 1. Introduction

University students are constantly subjected to stress in their daily life. It is a normal physical, mental, or emotional response to unexpected or unpleasant situations. A stressor is a personal or environmental factor that generates this response. Perceived stress is highly prevalent among undergraduate healthcare students, as reported in several studies [1,2,3,4,5,6]. Worldwide, perceived stress prevalence ranges from 31.0 to 63.5% among medical students [2,3]. In Saudi Arabia, studies conducted among Saudi medical students reported that more than half (59.2%) of medical students were found to be stressed [5,6,7]. In another Asian country, a study among 386 undergraduate medical students reported that severe and high level of stress was found among 61% of the students [8]. Short-term stress is usually manageable and beneficial as it increases students’ motivation for learning. However, persistent and severe student stress can lead to poor quality of life and well-being, burnout, and mental problems, such as anxiety and depression [9].

Anxiety is prevalent among medical students; the estimated global prevalence rate is 33.8% (i.e., one in three medical students suffer from anxiety), which is substantially higher than the general population, as reported in a systematic review of 69 studies comprising 40,348 medical students [10]. Anxiety was most prevalent among medical students from the Middle East and Asia [10]. Depression among healthcare students was higher than aged matched populations [11,12,13,14]. Worldwide, it affects around 27% of medical students [13,15], 34% of nurse students [14], and 30% of pharmacy students [16]. Both depression and anxiety have been linked to impaired functioning, burnout, poor academic performance, poor quality of life, and risk of suicidal behavior [15].

Although the association of perceived stress with depression and anxiety seems well established by many studies, the extent of perceived stress in healthcare students and its relation to mental health is neither fully understood and nor addressed in Saudi Arabia. Therefore, this study aims to investigate the level of perceived stress among healthcare students, evaluate the association between perceived stress and anxiety and depression, compare the perceived stress levels between different healthcare students, and identify factors associated with perceived stress to identify at-risk healthcare students and provide timely intervention. This study’s results should help decision-makers develop interventions to reduce stress among undergraduate healthcare students to maintain mental health and well-being.

## 2. Methods

### 2.1. Study Design and Setting

A cross-sectional study was conducted using an online structured questionnaire among healthcare students in Saudi Arabia. The data were collected from 26 September 2022 to 30 October 2022.

The questionnaire was disseminated anonymously across numerous multimedia platforms. The healthcare students were recruited using a snowball sampling technique. The survey was initially given to healthcare students, and they were urged to share the provided link with other students via various online platforms (such as WhatsApp, Twitter, student blogs, etc.). The offered link was shared widely and on various platforms in order to reduce the chance of selection bias. By clicking on a provided link, research participants could enroll by being redirected to the questionnaire.

### 2.2. Ethical Considerations and Consent to Participate

The study was approved by the Research Center of the Medical College of King Saud University and its Ethical Committee (Protocol No. E-21-6192). The informed consent was explicit and described the study goal and the participant’s freedom to withdraw at any time on the first page of the questionnaire. No participant identifiers were utilized in this study to ensure complete confidentiality, and all study participants remained anonymous.

### 2.3. Participants

The study sample was composed of undergraduate healthcare students from different healthcare schools. The inclusion criteria included undergraduate students of all levels of health science bachelor programs (medicine, pharmacy, dentistry, and nursing, applied medical sciences) who consented to participate in this study.

### 2.4. Questionnaire Development

An anonymous online structured questionnaire was developed after an extensive literature review. It contained the following four sections: (1) sociodemographic data (age, gender, nationality, and region of residence); (2) type of health science program and year of study; (3) current health status (health conditions and perceived physical health (excellent/very good, good, and fair/poor); and (4) perceived stress, depression, and anxiety. Then, a group of researchers (*n* = 4) reviewed the questionnaire for the relevance, content, and ease of understanding of the questions. The comments of the researchers were taken into account. Items included in the questionnaire covered all aspects of stress being measured and were linked to this study’s objectives.

### 2.5. Data Collection/Data Source

The questionnaire was written in the English language and hosted on a Google form. After IRB approval, the survey link was distributed online via email, Twitter, and WhatsApp. Participants’ consent was collected before participation. The survey cover letter included information about the study’s purpose and a statement that the participation was entirely voluntary and participants could withdraw at any time. Students who agreed to participate were asked to complete the questionnaires.

### 2.6. Variables

#### Outcome: Perceived Stress

Perceived stress was evaluated using the Perceived Stress Scale-14 (PSS), a widely used method for assessing psychological stress [17]. It is a self-reported questionnaire that aims to evaluate “the degree to which individuals perceive stressful situations in their lives” [18]. Each item is graded on a 5-point Likert scale, with 0 indicating “never” and 4 indicating “very often” [19]. PSS-14 scores were obtained by reversing the scores of seven positive items (4, 5, 6, 7, 9, 10, and 13), e.g., 0 = 4, 1 = 3, 2 = 2, 3 = 1, 4 = 0. After reversing the positive items’ scores and summing up all scores, the total scores range from 0 to 56. A score of 28 was used as a cutoff point between stressed and unstressed in previously published studies (i.e., a score of 0–28 points was considered unstressed, and a score above 28 was considered stressed).

### 2.7. Independent Variables

#### Key Independent Variables

Key independent variables include depression and anxiety. Depression and anxiety were measured using the Hospital Anxiety and Depression Scale (HADS). HADs is a self-assessment scale that was developed to assess the presence of depression and anxiety in patients [20]. The HADs scale was found to perform well in the general population and has been validated among undergraduates and medical students. HADS consists of 14 items, with 7 items each for the anxiety and depression subscales. Scores for each item range from 0 to 3. A subscale total score of 0–7 is considered normal; 8–10 indicates borderline depression or anxiety; and a score of 11 and over indicates a probable case of depression or anxiety.

### 2.8. Other Independent Variables

Independent variables included sociodemographic data (age, gender, nationality, and region of residence). The Kingdom of Saudi Arabia is divided into 13 emirates (provinces/regions), classified into 5 regions (East, West, Middle, North, and South). Other variables included health science program, year of study, current health status (health conditions, and perceived physical health (excellent/very good, good, and fair/poor)).

### 2.9. Sample Size

The estimated required sample size was 400 students using Sampsize; an online calculator. The prevalence of perceived stress was set at 50% with a 95% confidence interval and a 5% significance level.

### 2.10. Statistical Analysis

The analysis was performed using PSPP Statistical Analysis Software version 1.2.0. The categorical data statistics were presented as percentage and frequency, while the continuous data were presented as mean and standard deviation. Comparison between groups was evaluated using Chi-square tests and independent *t*-test. After adjusting for multiple confounders, a binary logistic regression analysis was conducted to examine the relationship between perceived stress and the independent variables. Adjusted odds ratios (AORs) with 95% confidence intervals (CIs) were calculated, and statistical significance was considered if *p*-value was (<0.05).

## 3. Results

### 3.1. Characteristics of the Study Sample

From a total of 950 students who viewed our online survey, after excluding incomplete answers, a total of 701 (73.8%) respondents filled out the form completely, and were included in the study. The average age of the students was 20.9 years old. Around 59.3% were female and 40.7% were male. Most of the students were from the middle region, 53.1%. The participants were from medical school (29.2%), followed by pharmacy (25.4%), applied medical science (21.1%), nursing (14.1%), and the dentistry school (10.1%). Around 10.8% of the participants reported having chronic health conditions. The majority of the participants (87.4%) perceived their physical health to be (excellent/very good/ good) (Table 1).

### 3.2. Perceived Stress among Healthcare Students

Almost three-quarters of the students were stressed (Table 1). A significantly higher level of perceived stress was reported among female students compared to males (*p* = 0.001) and those who reside in the West region (*p* = 0.022). No difference in perceived stress was observed between different healthcare programs. Perceived stress was reported to be significantly higher among third-year students (*p* = 0.048) and those who reported fair /poor perceived physical health (*p* < 0.001).

### 3.3. Association between Perceived Stress and Depression and Anxiety

Around 32.0% of the participants were categorized as having a probable case of depression, and 53.0% reported having a probable case of anxiety (Table 2).

There was a significant association between perceived stress, anxiety, and depression (Table 2). A significantly higher percentage of those with cases of anxiety perceived themselves as stressed compared to those without anxiety (86.0% vs. 39.9%, *p* value < 0.001). Likewise, a significantly higher percentage of those with cases of depression perceived themselves as stressed compared to those without depression (89.8% vs. 52.0%, *p* value < 0.001).

### 3.4. Factors Associated with Perceived Stress from Adjusted Regression Analysis

A multiple adjusted regression analysis was used to identify independent factors associated with perceived stress (Table 3). Female students [Adjusted Odds Ratio (AOR) = 1.66; 95% CI 1.06–2.61] were more likely to perceive themselves as stressed. Perceived stress was four times more likely among students with cases of anxiety [AOR = 4.83; 95% CI 2.89, 8.06], depression [AOR = 4.79; 95% CI 2.68, 8.53] as compared with those without these conditions. Students from the East region [AOR= 0.39; 95% CI 0.18–0.86] were less likely to perceive themselves as stressed.

## 4. Discussion

The study found that perceived stress was highly prevalent among healthcare students in Saudi Arabia. Mental health is an important factor that affects the odds of perceived stress. Our findings of the higher prevalence of perceived stress, almost two-thirds, are consistent with previously published studies among medical students in Saudi Arabia [5,6,21], however, are higher than those that have been reported worldwide (31.0–63.5%) among medical students [2,3].

A critical study finding was the strong association between perceived stress and probable cases of anxiety and depression. This finding aligns with previous studies underlining this significant relationship [22,23]. Our study added to the literature that this association exists among students in different healthcare schools. This study’s findings can be used to indicate student psychological well-being and as a baseline for future research. Students must be aware of stress management and coping techniques to cope with difficult situations. This suggests that universities should give more attention to healthcare students’ psychological well-being; hence, stress management programs and support systems should be offered to healthcare students.

Other factors were associated with perceived stress in this study. Female students reported higher perceived stress than male students, which has also been documented in prior studies [24,25,26]. These gender-specific disparities in the prevalence of perceived stress may result from the different ways that men and women process emotions and are exposed to stressful situations, as well as from sociocultural and biological variables. The participant’s perception of stress was compared between study years. Our findings indicate that students in all five programs do not have a statistically significant difference in perception of stress from the adjusted regression analysis; this was also reported in one other study [27]. Although students in the third year showed higher perceived stress, students in other years of study also have a high level of perceived stress; this could be explained by students who are in the higher years of their study may become more adapted to university environments, and as a result, they are more prepared to deal with stress than students in their early years. Moreover, students with fair or poor physical health were more likely to perceive stress than those with excellent physical health. The results agree with a study that found an association between stress and students’ self-reported physical problems [26]. Physical activity can enhance a person’s physical health and provide long-term physical health benefits in high perceived stress individuals [28]. In addition, a Mindfulness Program over the course of 16 weeks has been shown to be helpful in reducing perceived stress in medical students [29].

### 4.1. Implications

The study’s conclusions may have a big impact on how medical schools should handle stress. In healthcare schools, there has to be a greater emphasis on mental health awareness to boost students’ academic happiness, overall well-being, and academic achievement early on. Additionally, it is crucial that any programs offered to minimize the negative impacts of stress be expanded by student welfare services. It has been proven that a variety of techniques can help college students feel less stressed. The therapeutic usefulness of cognitive-behavioral therapy, coping mechanisms, and social support measures in reducing stress among university populations is underlined by a thorough analysis of stress management techniques targeted at university students.

### 4.2. Strength/Limitations

This study included multiple universities from different regions in Saudi Arabia, which makes the results more generalizable to Saudi healthcare students. Most previous studies focused on one healthcare discipline; in contrast, this study compared perceived stress across different healthcare programs. Some limitations of the present study should be considered. The cross-sectional design is not able to evaluate the causal relationship. Additionally, other factors, such as financial status and academic performance, should have been addressed. Finally, we cannot rule out response bias since the data were collected using a self-administered questionnaire. Students may have overestimated or underestimated their stress and feelings. Another limitation of this study is that test–retest reliability was not undertaken in order to determine the reliability of the administered questionnaire. Additionally, PSS and HADS scales have not been validated among healthcare students in Saudi Arabia.

## 5. Conclusions

Perceived stress was highly prevalent among healthcare students in Saudi Arabia; almost two-thirds of healthcare students have stress. Female gender, anxiety, and depression have been significant risk factors for perceived stress. A healthcare student’s mental health is an essential factor affecting the perceived stress level and at-risk individuals. Therefore, preventative mental health interventions targeting healthcare students are crucial to help improve mental health and cope with stressors in academic education.

## Figures and Tables

**Table 1:** Characteristics of the study sample, healthcare students.

	Total		Not Stressed		Stressed				
	N	%	N	%	N	%	Chi-Square Value	*p* Value	Sig.
	701	100.0	192	27.4	509	72.6			
**Age Mean (SD)**	20.94 ± 1.92		21.05 ± 1.81		20.9 ± 1.96		16.07	0.372	
**Gender**							10.66	0.001	**
Male	285	40.7	97	34.0	188	66.0			
Female	416	59.3	95	22.8	321	77.2			
**Region of Residence**							11.41	0.022	*
North	76	10.8	21	27.6	55	72.4			
East	85	12.1	36	42.4	49	57.7			
Middle	372	53.1	93	25.0	279	75.0			
South	62	8.8	17	27.4	45	72.6			
West	106	15.1	25	23.6	81	76.4			
**Health Science Program**							2.0	0.735	
Medicine	205	29.2	58	28.3	147	71.7			
Dentistry	71	10.1	19	26.8	52	73.2			
Pharmacy	178	25.4	45	25.3	133	74.7			
Nursing	99	14.1	24	24.2	75	75.8			
Applied Medical Science	148	21.1	46	31.1	102	68.9			
**Year of Study**							12.71	0.048	*
First Year	89	12.7	24	27.0	65	73.0			
Second Year	171	24.4	40	23.4	131	76.6			
Third year	85	12.1	13	15.3	72	84.7			
Fourth Year	127	18.1	41	32.3	86	67.7			
Fifth Year	82	11.7	24	29.3	58	70.7			
Sixth Year	50	7.1	16	32.0	34	68.0			
Internship	97	13.8	34	35.1	63	65.0			
**Chronic Health Condition**							1.72	0.190	
Yes	76	10.8	16	21.1	60	79.0			
No	625	89.2	176	28.2	449	71.8			
**Perceived Health Condition**							31.27	<0.001	***
Excellent	154	22.0	67	43.5	87	56.5			
Very Good	222	31.7	61	27.5	161	72.5			
Good	237	33.8	51	21.5	186	78.5			
Fair or Poor	88	12.6	13	14.8	75	85.2			

Note: The study sample comprised of 701 healthcare students. Asterisks (*) represent significant differences in Perceived stress from chi-square tests. *** *p* < 0.001; ** 0.001 ≤ *p* < 0.01; * 0.01 ≤ *p* < 0.05.

**Table 2:** Perceived stress of healthcare students depending on depression and anxiety.

	Total		Not Stressed		Stressed				
	N	%	N	%	N	%	Chi-Square Value	*p* Value	Sig.
	701	100.0	192	27.4	509	72.6			
**Anxiety**							124.75	<0.001	***
Normal	168	24.0	101	60.1	67	39.9			
Borderline	162	23.1	39	24.1	123	75.9			
Case	371	52.9	52	14.0	319	86.0			
**Depression**							91.43	<0.001	***
Normal	252	36.0	121	48.0	131	52.0			
Borderline	223	31.8	48	21.5	175	78.5			
Case	226	32.2	23	10.2	203	89.8			

Note: The study sample comprised of 701 healthcare students. Asterisks (*) represent significant differences in Perceived stress from chi-square tests. *** *p* < 0.001.

**Table 3:** Adjusted Odds Ratios and 95% Confidence Intervals from Logistic Regression on Perceived Stress.

	AOR	95% CI	*p* Value	Sig.
**Age Mean (SD)**	1.27	[1.03, 1.58]	0.027	*
**Gender**			0.028	*
Female	1.66	[1.06–2.61]		
Male **(Ref.)**				
**Region of Residence**			0.039	*
West	1.19	[0.52, 2.71]		
East	0.39	[0.18, 0.86]		
Middle	0.96	[0.50, 1.83]		
South	0.70	[0.29, 1.69]		
North **(Ref.)**				
**Health Science Program**			0.94	
Medicine	1.03	[0.56, 1.89]		
Dentistry	1.22	[0.53, 2.81]		
Nursing	1.02	[0.47, 2.23]		
Applied Medical Science	0.85	[0.47, 1.54]		
Pharmacy **(Ref.)**				
**Year of Study**			0.015	*
First Year	0.42	[0.15, 1.17]		
Second Year	0.50	[0.21, 1.19]		
Fourth Year	0.19	[0.08, 0.46]		
Fifth Year	0.26	[0.09, 0.74]		
Sixth Year	0.24	[0.07, 0.85]		
Internship	0.16	[0.05, 0.49]		
Third year **(Ref.)**				
**Chronic Health Condition**			0.597	
No	0.83	[0.42, 1.65]		
Yes **(Ref.)**				
**Perceived Health Condition**			0.018	*
Excellent	0.66	[0.39, 1.12]		
Good	1.40	[0.84, 2.35]		
Fair or poor	1.90	[0.87, 4.14]		
Very Good **(Ref.)**				
**Anxiety**			<0.001	***
Borderline	3.42	[2.10, 5.84]		
Case	4.83	[2.89, 8.06]		
Normal **(Ref.)**				
**Depression**			<0.001	***
Borderline	2.25	[1.38, 3.66]		
Case	4.79	[2.68, 8.53]		
Normal **(Ref.)**				

The study sample comprised of 701 healthcare students. AOR: Adjusted Odds Ratio; CI: Confidence Interval; Ref: Reference Group. Asterisks (*) represent significant differences based on perceived stress. *** *p* < 0.001; * 0.01 ≤ *p* < 0.05.

## Data Availability

The data used to support the findings of this study are available from the corresponding authors upon request.
